# An Unusual Aquatic Eruption

**DOI:** 10.7759/cureus.81809

**Published:** 2025-04-06

**Authors:** Kimberly Boerner, Anita Bachu, Richard Rubenstein

**Affiliations:** 1 Dermatology, Skin Science, Phoenix, USA; 2 Dermatology, Larkin Community Hospital Palm Springs Campus, Hialeah, USA

**Keywords:** aquatic, aquatic infections, dermatitis, fish handler's disease, jellyfish, mycobacterium marinum, sea bather's eruption, seaweed dermatitis, swimmer's itch

## Abstract

As more people come into contact with seawater and freshwater bodies each year, medical providers are increasingly likely to encounter cases of aquatic dermatitis. The cutaneous manifestations caused by marine organisms can range from a pruritic, macular rash to a painful, vesicular eruption. Some conditions, such as those resulting from jellyfish envenomation, present with a distinct pattern allowing precise diagnosis and treatment. However, other conditions, such as swimmer’s itch, may cause vague dermatitis, making diagnosis more challenging. Determining the etiology can aid in the overall management and treatment of these patients.

## Introduction

Aquatic eruptions are increasingly recognized as an unusual form of dermatitis. These reactions can result from various marine exposures, including swimmer's itch (cercarial dermatitis) and sea bather’s eruption (caused by sea anemone and thimble jellyfish). Other etiologies include seaweed dermatitis, *Mycobacterium marinum* infection, and erysipeloid (fish handler’s disease). Clinical presentations are often variable, which can complicate definitive diagnosis. However, the morphology, distribution, and associated symptoms of the eruption can provide valuable diagnostic clues. Microbiologic cultures and histopathologic examination may further support the diagnosis. Here, we present a case of an unusual cutaneous eruption in a previously healthy man following ocean exposure off the coast of Florida. We also review the differential diagnosis and diagnostic approach to aquatic dermatitis.

## Case presentation

A 44-year-old Caucasian male patient presented to the dermatology clinic with a three-day history of a vesiculobullous skin eruption affecting the left dorsal hand, lower legs, and back. The patient described the rash as pruritic and burning but not painful. The eruption initially appeared on the dorsum of the left hand and later spread to the lower legs and back. He denied systemic symptoms, including fever, chills, and gastrointestinal distress. 

The patient reported swimming in the ocean and handling fish during a fishing excursion one day before the rash onset. He wore standard men’s swim trunks during these activities. Notably, none of his companions, who were in direct contact with him while fishing and swimming, developed similar symptoms.

At symptom onset, the patient initially sought care at an urgent care clinic. Concerned about necrotizing fasciitis, he was referred to the emergency department (ED) for further evaluation. In the ED, the diagnosis of necrotizing fasciitis was deemed unlikely due to the absence of systemic symptoms, lack of severe pain, and limited lesion spread. He was empirically prescribed oral cephalexin and doxycycline, which he began the same day.

Three days later, the patient presented to our dermatology clinic with persistent lesions but no evidence of progression. He remained afebrile and reported no new systemic symptoms. His past medical history was unremarkable, and he was not taking any medications other than those prescribed in the ED.

**Figure 1:**
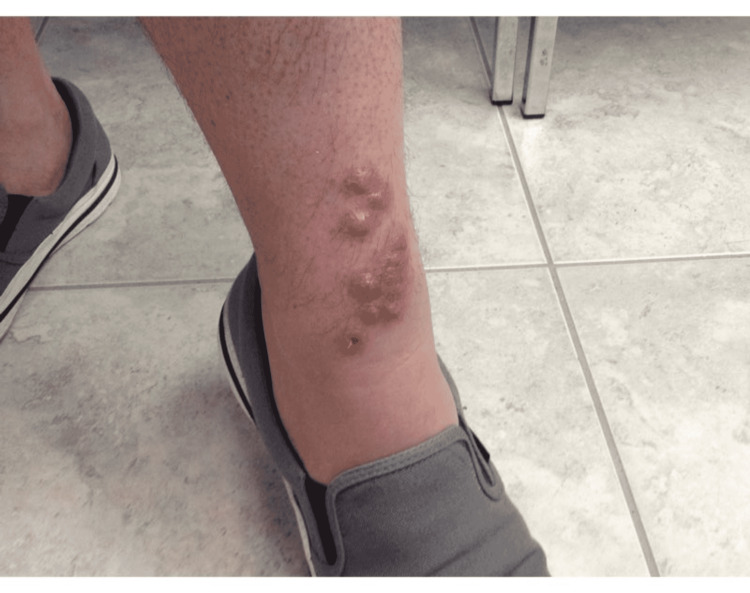
Left lower leg showing grouped pink/red erythematous nodules with ulceration

**Figure 2:**
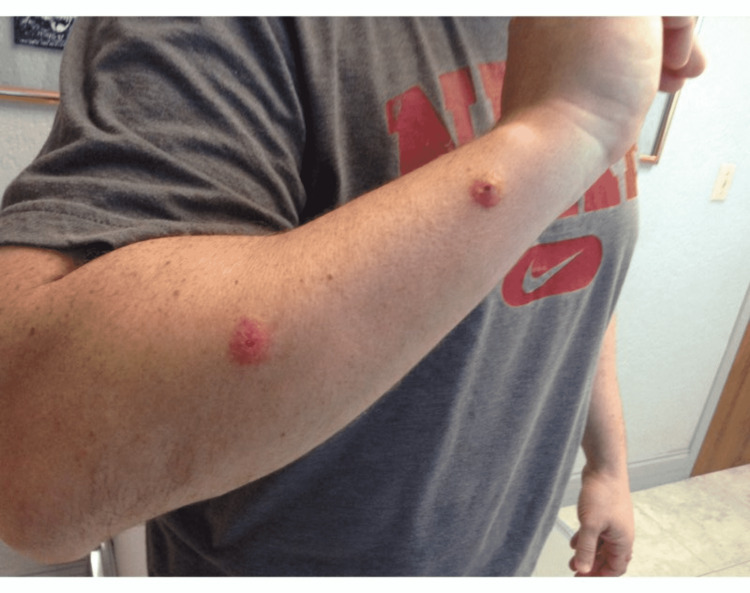
Two well-demarcated pink erythematous nodules with ulceration on the right forearm

**Figure 3:**
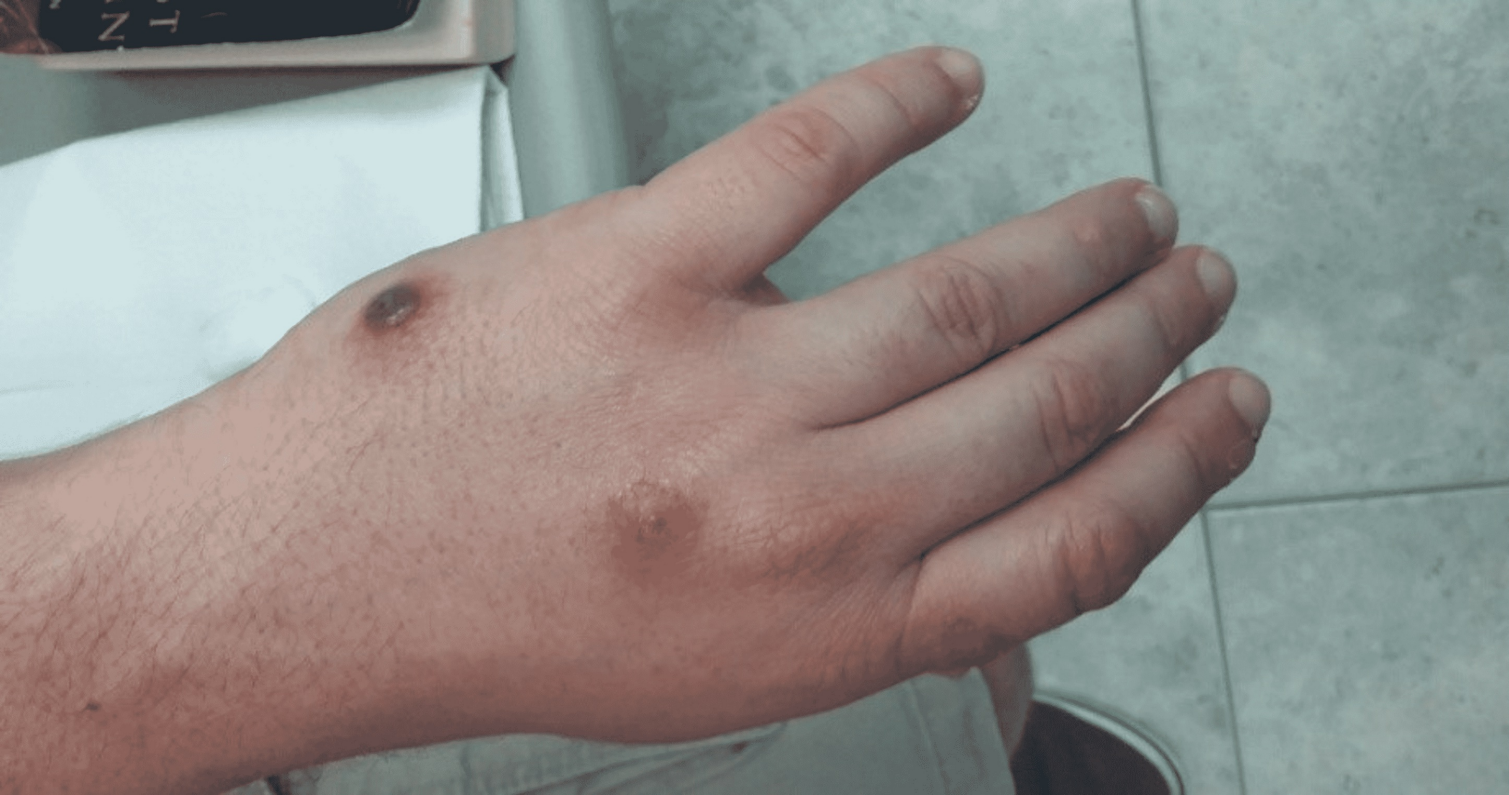
Two well-demarcated reddish brown nodules with ulceration and surrounding erythema, located on the dorsal aspect of the left hand

**Figure 4:**
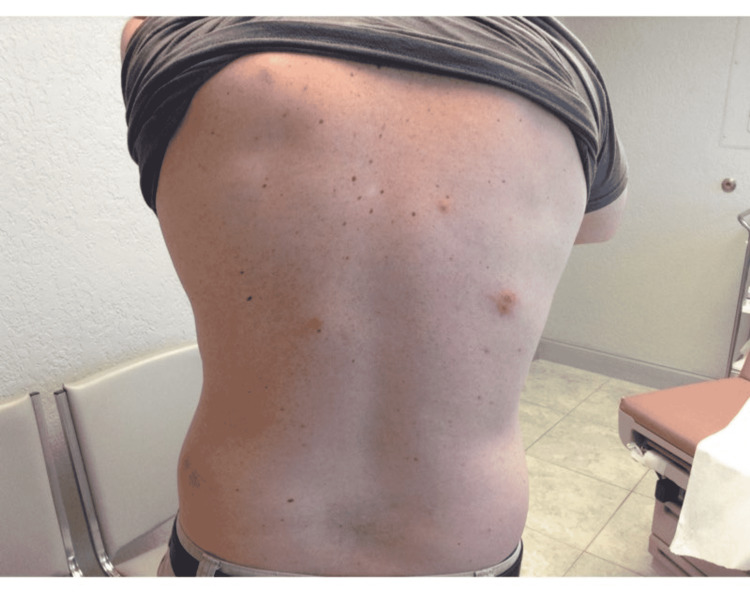
Three pink erythematous nodules on the patient's back

Physical examination revealed multiple well-demarcated, pink erythematous bullae and nodules localized to the ulnar aspect of the dorsum of the left hand, the left lower leg, and back (Figures 1-4). No additional lesions were noted. A shave biopsy and bacterial culture were performed on a representative lesion from the left lower leg.

The patient was advised to continue his oral antibiotic regimen and prescribed a six-day tapering course of methylprednisolone (24 mg on day 1, with 4 mg reduction per day). Additionally, he was instructed to apply Burow’s solution compresses twice daily, followed by topical gentamicin 0.1% ointment.

The patient returned to our dermatology clinic one week later for a follow-up evaluation. Examination revealed marked clinical improvement, with no new lesions. By the three-week mark, the eruption had completely resolved. 

**Figure 5:**
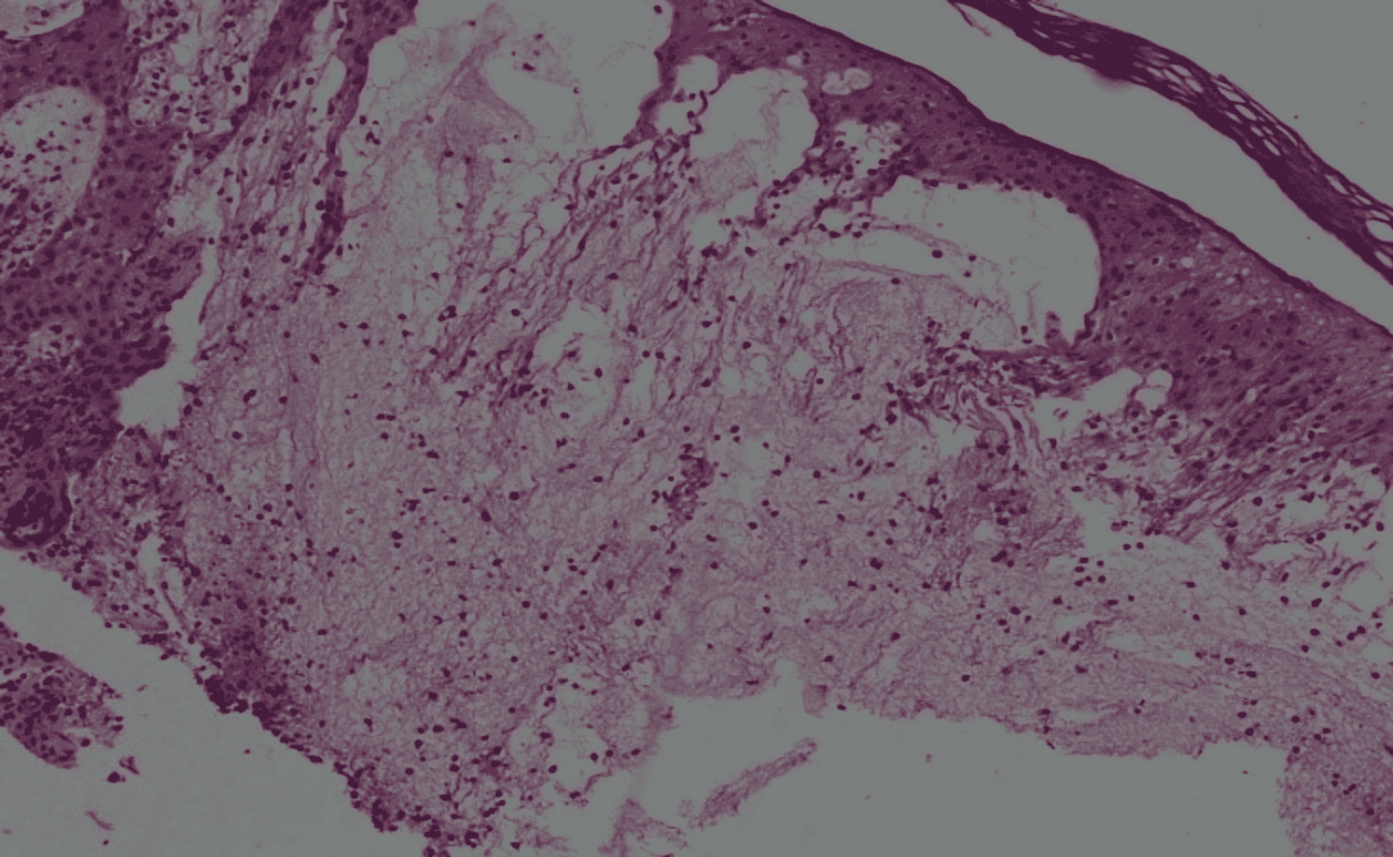
Histopathology sample of a left lower leg cutaneous nodule demonstrating marked dermal edema

**Figure 6:**
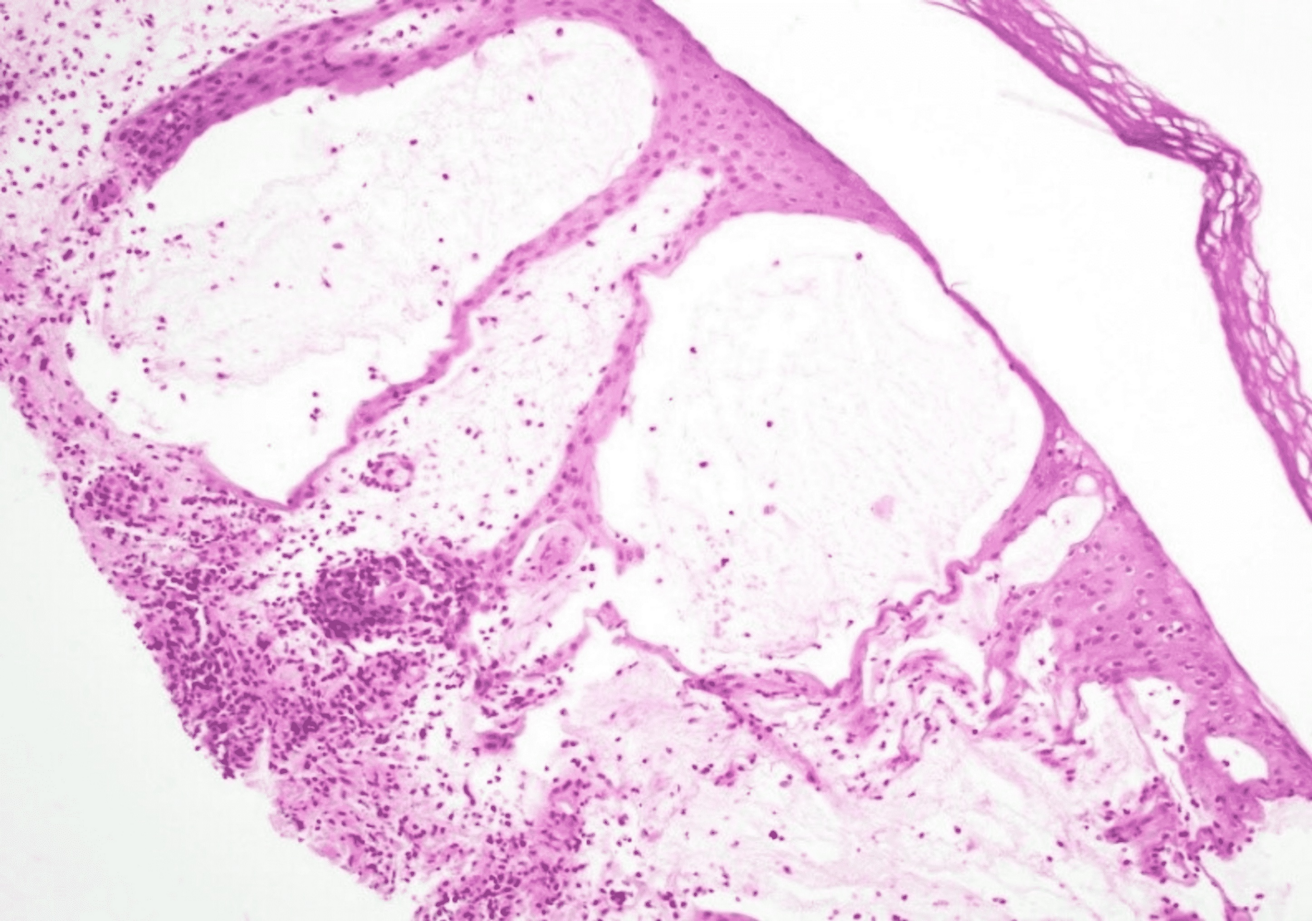
Histopathology sample of a left lower leg cutaneous nodule revealing a mixed cell infiltrate of lymphocytes, neutrophils, and eosinophils

Histopathology showed a marked dermal edema and a mixed-cell infiltrate consisting of lymphocytes, neutrophils, and eosinophils (Figure 5 and Figure 6). Bacterial cultures and acid-fast bacillus (AFB) testing were negative.

## Discussion

This case represents an unusual marine-induced cutaneous eruption with a broad differential diagnosis. Although no single etiology was definitively identified, the eruption was self-limited and resolved completely with supportive care. A review of the literature reveals several marine-associated dermatoses that could explain the clinical findings observed in this patient.

Sea bather’s eruption

Sea bather’s eruption is a pruritic dermatitis resulting from a hypersensitivity reaction to marine organisms, most commonly the larvae of the thimble jellyfish (*Linuche unguiculata*) [1,2] and, in some cases, the larval form of the sea anemone *Edwardsiella lineata* [3,4]. The condition is sometimes colloquially referred to as "sea lice"; however, this term is discouraged, as true sea lice are parasitic copepods that affect fish [5]. First reported in 1949 off the eastern coast of Florida, sea bather’s eruption is now recognized in the Caribbean, coastal Mexico, the Southern United States, and parts of South America [6].

The eruption is believed to occur when jellyfish larvae become trapped beneath swimwear, particularly after prolonged seawater exposure. Patients typically develop pruritus within hours of exposure, with possible systemic symptoms including fever, malaise, abdominal pain, nausea, vomiting, and diarrhea. Physical examination often reveals inflammatory papules corresponding to areas covered by swimwear. Diagnosis is clinical, as histopathologic findings are nonspecific. When performed, biopsy typically reveals a superficial and deep perivascular and interstitial infiltrate composed of lymphocytes, eosinophils, and neutrophils, with preservation of the epidermal-dermal junction and no significant epidermal changes [1]. Treatment is supportive, with high-potency topical corticosteroids and oral antihistamines. Systemic corticosteroids may be necessary in more severe cases. Lesions generally resolve within one week [7,8].

Swimmer's itch (cercarial dermatitis)

Swimmer’s itch, or cercarial dermatitis, is a hypersensitivity reaction caused by skin penetration of schistosome larvae (cercariae) released from infected freshwater and saltwater snails. The natural hosts are typically birds and nonhuman mammals; humans are incidental hosts [9]. Upon contact with the human skin, the cercariae burrow into the epidermis, triggering an allergic response. A macular rash appears within 24 hours, often progressing to a maculopapular or vesicular eruption [10]. As humans are not suitable hosts, the organisms do not survive, and the condition is self-limiting, typically resolving within 2-3 days.

Diagnosis is based on history of exposure to potentially contaminated waters. Treatment is symptomatic, involving topical antipruritics, antihistamines, and mild corticosteroids in more severe cases. Swimmer’s itch can be differentiated from sea bather’s eruption by its distribution: the former typically affects the exposed skin, while the latter involves areas covered by tight-fitting garments [9].

*Mycobacterium marinum *infection

*Mycobacterium marinum* is an atypical mycobacterial species found in a range of aquatic environments, including both fresh- and saltwater sources. It is known to cause granulomatous skin infections, often presenting as papules, nodules, or ulcerative lesions at the site of inoculation, sometimes with lymphangitic spread. The clinical presentation may mimic interstitial granuloma annulare [11,12].

Infections are often associated with exposure to aquariums, fishing equipment, or marine animals. The incidence is estimated at 0.27 cases per 100,000 adults annually, and immunocompromised individuals are at increased risk of disseminated infection, including osteomyelitis and septic arthritis. Lesions frequently occur on the cooler extremities, particularly the upper limbs, due to the organism’s preference for temperatures around 32°C [11]. The incubation period ranges from a few weeks to several months [13]. Treatment typically involves prolonged antibiotic therapy (2 weeks to 18 months), with agents such as rifampin, ethambutol, minocycline, TMP-SMX, clarithromycin, or ciprofloxacin. Heat therapy with hot compresses may aid treatment, as the organism is heat-sensitive [6].

Seaweed dermatitis

Seaweed dermatitis is an irritant contact dermatitis caused by exposure to *Lyngbya majuscula*, a cyanobacterium found in warm marine environments such as those off the coasts of Florida and Hawaii. The cyanobacterium produces toxins including lyngbyatoxin A and debromoaplysiatoxin, which can cause skin irritation within 24 hours of exposure [14]. Patients typically report burning or stinging sensations followed by erythematous blisters and desquamation, often affecting the genital, perineal, and perianal areas. Pruritus is common. Symptoms generally resolve within several days to a week. Treatment is supportive and includes topical corticosteroids, antihistamines, and analgesics. Inhalation of the toxin may also cause respiratory and gastrointestinal irritation [14,15].

Erysipeloid (fish handler's disease)

Erysipeloid, or fish handler’s disease, is caused by *Erysipelothrix rhusiopathiae*, a Gram-positive facultative anaerobe found in both fresh and salt water. The bacterium enters through skin abrasions and causes a localized violaceous plaque with a raised border, most often on the hands and between the fingers [13]. There are three clinical forms: localized cutaneous, diffuse cutaneous, and systemic. The diffuse form presents with multiple skin lesions, while the systemic form may cause endocarditis and lymphadenopathy without cutaneous manifestations [16].

Histopathologic findings include epidermal spongiosis, papillary dermal edema, and a perivascular lymphocytic infiltrate in the reticular dermis. Diagnosis is confirmed by skin biopsy and culture. First-line treatment includes penicillin, erythromycin, or doxycycline; the organism is resistant to sulfonamides and vancomycin [13].

## Conclusions

This case highlights an unusual aquatic-associated eruption characterized by the acute onset of cutaneous vesiculobullae and nodules. Clinical and histopathologic examination revealed a mixed-cell dermatitis consistent with a marine-related etiology. Awareness of the diverse spectrum of aquatic dermatoses is essential for clinicians, particularly in coastal and tropical regions. This case also underscores the diagnostic challenge posed by marine-related cutaneous eruptions. Although the precise etiology of this patient was not identified, several plausible differentials, including sea bather’s eruption, swimmer's itch, and seaweed dermatitis, were considered. The eruption was ultimately self-limited and resolved with symptomatic therapy.
